# Authors’ Reply: Clarifying the Comparative Interpretation and Clinical Implications of Radiomics-Based AI for Pathological Response Prediction

**DOI:** 10.2196/110607

**Published:** 2026-10-06

**Authors:** Ziqi Jiang, Yuan Xu, Shuyu Jia, Hongsheng Liu

**Affiliations:** 1 Department of Thoracic Surgery, Peking Union Medical College Hospital, Chinese Academy of Medical Science, 1 Shuaifuyuan, Wangfujing, Beijing, 100730, China, 86 13621021237; 2 Department of Infectious Diseases, Nanfang Hospital, Southern Medical University, Guangzhou, China

**Keywords:** radiomics, non–small cell lung carcinoma, neoadjuvant therapy, pathological response, meta-analysis, NSCLC

We thank the correspondents [1] for their careful reading of our systematic review and meta-analysis [2]; the issues raised are addressed below. We agree that Table S11 needs clarification: RECIST 1.1 (Response Evaluation Criteria in Solid Tumors version 1.1) and PERCIST (Positron Emission Tomography Response Criteria in Solid Tumors) were evaluated in the same 36 patients by Deng et al [3], so the reported number 72 represents 72 test assessments, not 72 unique participants. Because only validation cohorts entered our pooled analysis, Deng et al’s [3] 8-patient external-validation cohort contributed to the AI synthesis, whereas the RECIST 1.1 and PERCIST results came from the 36-patient development cohort; the *Z* test thus compared different evidence sets and should be interpreted as a cross-study, not head-to-head, comparison.

We also agree that the pattern reflects a sensitivity-specificity trade-off rather than uniform superiority: for pathological complete response (pCR), AI showed higher sensitivity and conventional criteria higher specificity, so greater sensitivity alone does not imply overall superiority. Notably, no same-patient head-to-head study is currently available; in the single direct comparison, Deng et al [3] reported sensitivity and specificity of 100.0% and 94.1%, respectively, for the prediction model, 10.5% and 100.0% for RECIST 1.1, and 73.7% and 94.1% for PERCIST, which is directionally consistent with our findings, although the values were excluded from the pooled analysis. These limitations therefore affect the strength of the comparative-superiority inference rather than the principal finding that radiomics-based AI shows promising diagnostic performance.

Our eligibility criteria required postoperative pathological assessment (pCR and major pathological response [MPR] as targets, surgical histopathology as reference standard), so the pooled estimates apply to patients that underwent a resection and should not be extrapolated to an intention-to-treat population. Prediction timing is another source of heterogeneity, as models used pretreatment, during-treatment, and preoperative or longitudinal imaging; pooling therefore characterizes overall performance but cannot establish the optimal decision time point. As in the CheckMate 816 trial [4], future studies should define whether pathological response applies to the primary tumor, nodes, or both, and incorporate nonsurgical patients.

We also acknowledge 2 errors in the Discussion section: a 25% diameter reduction is stable disease, not a partial response (which requires ≥30%) [5], and fibrosis misread as residual tumor yields a false negative, not a false positive, when pCR or MPR is positive; these errors do not affect the contingency data, pooled estimates, or primary analyses. The posttest probabilities likewise do not demonstrate readiness to determine surgical timing, de-escalation, or organ preservation; clinical implementation requires multicenter validation, locked models and thresholds, standardized time points, same-patient comparison, calibration, decision-curve analysis, and patient-relevant outcomes, consistent with DECIDE-AI [6].

In summary, the central finding is unchanged: radiomics-based AI shows promising performance for predicting pathological response after neoadjuvant immunochemotherapy among patients that underwent a resection, with a possible sensitivity advantage over RECIST 1.1; definitive superiority and treatment-changing utility require same-patient, same–time point studies.

## Abbreviations

MPR: major pathological response
pCR: pathological complete response
PERCIST: Positron Emission Tomography Response Criteria in Solid Tumors
RECIST: Response Evaluation Criteria in Solid Tumors
